# Dormancy cycling: translation‐related transcripts are the main difference between dormant and non‐dormant seeds in the field

**DOI:** 10.1111/tpj.14626

**Published:** 2020-02-05

**Authors:** Gonda Buijs, Afke Vogelzang, Harm Nijveen, Leónie Bentsink

**Affiliations:** ^1^ Wageningen Seed Laboratory Laboratory of Plant Physiology Wageningen University Wageningen the Netherlands; ^2^ Bioinformatics Group Wageningen University Wageningen the Netherlands

**Keywords:** *Arabidopsis thaliana*, *DELAY OF GERMINATION*, dormancy cycling, field experiment, secondary dormancy, seed biology, transcriptomics

## Abstract

Primary seed dormancy is a mechanism that orchestrates the timing of seed germination in order to prevent out‐of‐season germination. Secondary dormancy can be induced in imbibed seeds when they encounter prolonged unfavourable conditions. Secondary dormancy is not induced during dry storage, and therefore the mechanisms underlying this process have remained largely unexplored. Here, a 2‐year seed burial experiment in which dormancy cycling was studied at the physiological and transcriptional level is presented. For these analyses six different *Arabidopsis thaliana* genotypes were used: Landsberg *erecta* (L*er*) and the dormancy associated *DELAY OF GERMINATION* (*DOG*) near‐isogenic lines 1, 2, 3, 6 and 22 (NIL*DOG1*, *2*, *3*, *6* and *22*). The germination potential of seeds exhumed from the field showed that these seeds go through dormancy cycling and that the dynamics of this cycling is genotype dependent. RNA‐seq analysis revealed large transcriptional changes during dormancy cycling, especially at the time points preceding shifts in dormancy status. Dormancy cycling is driven by soil temperature and the endosperm is important in the perception of the environment. Genes that are upregulated in the low‐ to non‐dormant stages are enriched for genes involved in translation, indicating that the non‐dormant seeds are prepared for rapid seed germination.

## Significance Statement

Little is known about seed dormancy under natural conditions. This field study provides an in‐depth insight, at the physiological and transcriptional level, of how Arabidopsis seeds cycle through dormancy. We show that dormancy is strongly regulated by the environment, both by seasonal changes as well as by sudden changes. We identified large transcriptional changes and show that in particular the translational machinery is upregulated when seeds are non‐dormant.

## Introduction

Seed germination must be precisely timed to ensure the right conditions for a seedling to survive. Seed dormancy is the temporal inability of a viable seed to germinate under conditions that permit germination, and is the mechanism that regulates germination in the right season. Seed dormancy is regulated by an interplay between genetic factors and the environment (e.g. temperature)*.* The level of dormancy in a seed at the moment of shedding from the mother plant is called primary dormancy. Natural accessions originating from different habitats of *Arabidopsis thaliana* exhibit great variation in their primary dormancy phenotypes. Genetic studies employing this natural variation identified the *DELAY OF GERMINATION* (*DOG*) loci after quantitative trait loci analyses for primary seed dormancy release by dry after‐ripening (AR) (Alonso‐Blanco *et al.*, 2003). The DOG1 protein was identified and characterized as a major regulator of seed dormancy (Bentsink *et al.*, 2006; Nakabayashi *et al.*, 2012; Cyrek *et al.*, 2016; Née *et al.*, 2017). Key to regulating dormancy within the seeds are the plant hormones ABA (promoting dormancy) and GA (promoting germination). Not only the absolute levels of these two hormones but also the balance between the two determine the start of germination (Baskin and Baskin, 2004; Bewley *et al.*, 2013). Cold stratification releases dormancy from seeds by rapidly decreasing the levels of ABA in dormant seeds (reviewed by Vishal and Kumar, 2018).

The environment has a major effect on the establishment of dormancy, both during seed development and when seeds have entered the soil seed bank. Different maternal growth conditions can result in variable dormancy phenotypes of the same genotype (He *et al.*, 2014; Springthorpe and Penfield, 2015; Footitt *et al.*, 2019). Seeds that do not germinate can enter the soil seed bank where secondary dormancy can be induced if seeds experience long periods of unfavourable conditions (Barazani *et al.*, 2012). In seeds of genotypes with high primary dormancy levels, secondary dormancy is induced more rapidly (Coughlan *et al.*, 2017). The extent to which the mechanisms underlying primary dormancy also regulate secondary dormancy is unclear, although some of the mechanisms do overlap. A number of genes that are related to primary dormancy have been investigated during secondary dormancy cycling in the soil (Footitt *et al.*, 2011). *DOG1* and *MOTHER OF FLOWERING TIME* (*MFT*) expression, both inhibitors of germination, are highly expressed in secondary dormant seeds. Gene expression of the ABA catabolism gene *CYTOCHROME P450*, *FAMILY 707*, *SUBFAMILY A*, *POLYPEPTIDE 2* (*CYP707A2*) was low in secondary dormant seeds (Footitt *et al.*, 2011).

Different structures of the seed have specific functions in the regulation of germination and dormancy (Baskin and Baskin, 2004; Bewley *et al.*, 2013). Arabidopsis seeds consist of a relatively large embryo, surrounded by a single‐cell layer of endosperm and a seed coat (testa). Arabidopsis seeds have coat‐enhanced, non‐deep physiological dormancy: at maturity the embryo is fully developed but internal signals prevent germination, and this dormancy can be enhanced by the layers surrounding the embryo (Debeaujon and Koornneef, 2000; Lefebvre *et al.*, 2006; Graeber *et al.*, 2014): as a result of the production of ABA by the endosperm, for example (Lefebvre *et al.*, 2006; Lee *et al.*, 2010). The embryo also produces ABA, but the dormancy state of the endosperm overrules that of the embryo (Penfield *et al.*, 2010; Lee *et al.*, 2010). Recent research shows that some primary dormancy‐related genes are regulated through the epigenetic regulation of endosperm‐specific gene expression (Piskurewicz *et al.*, 2016; Iwasaki *et al.*, 2019).

**Figure 1:**
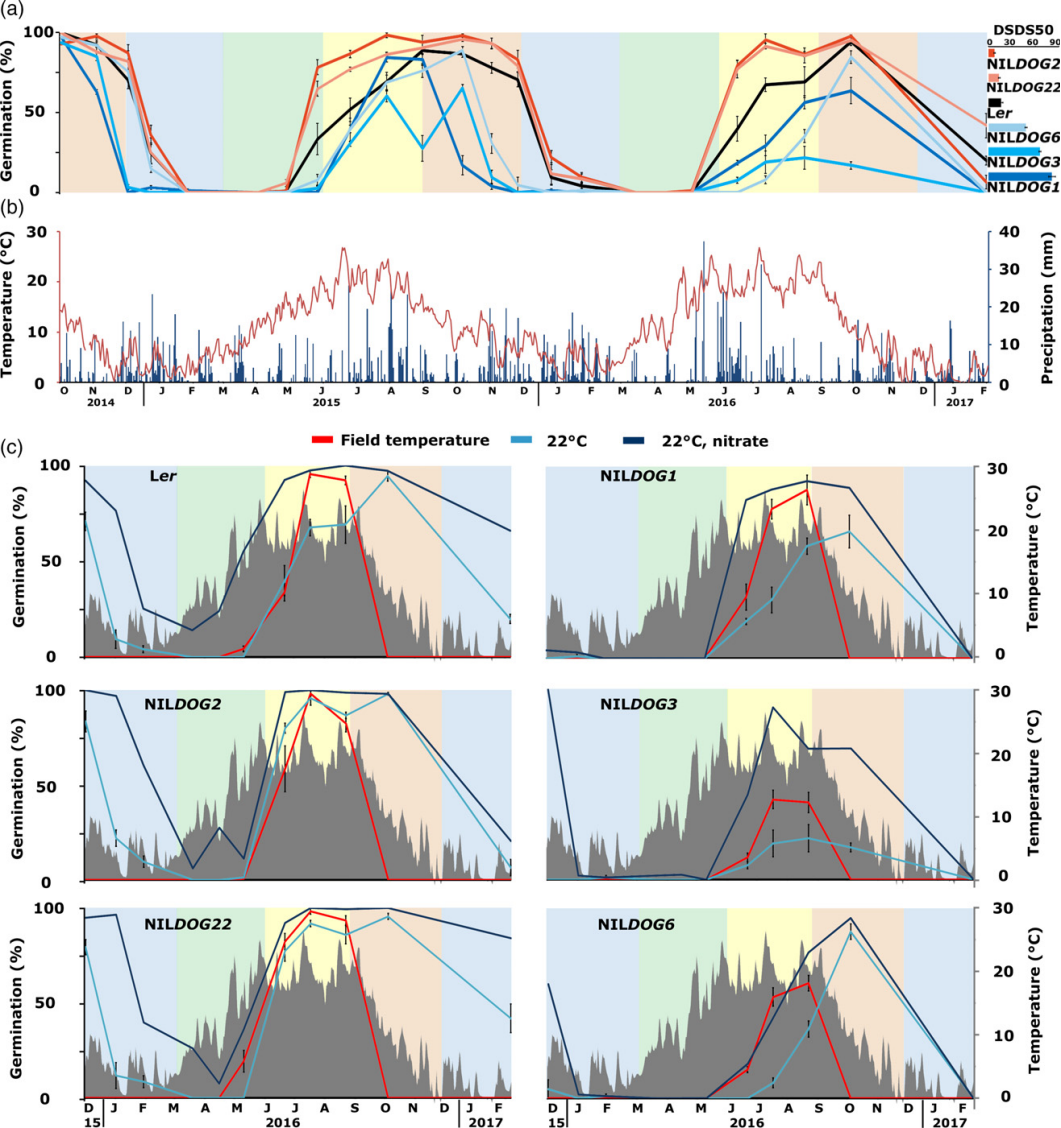
Dormancy cycling throughout the seasons. (a) Germination percentages of the six genotypes (four replicates per genotype). Seeds were exhumed from the field and analysed in the laboratory. The bar chart to the right indicates the primary dormancy levels of these genotypes, expressed as DSDS50 (days of seeds dry storage required to reach 50% germination) values. (b) The temperature of the soil at 5 cm depth (red, data from buried sensors) and the precipitation in mm (blue) at the time of the field experiment. (c) The germination capacity of the six genotypes when germinated at 22°C (blue), at 22°C with additional nitrate (deep blue) and at the outside temperature (red) at the moment of exhumation. The temperature in the soil over time is shown in grey. Coloured blocks indicate the seasons: blue, winter; green, spring; yellow, summer; and red, autumn.

Studies investigating the regulation of seed dormancy have mostly been performed under laboratory conditions, even those considering secondary dormancy. Here we study dormancy cycling under field conditions, using five near‐isogenic lines of the *DELAY OF GERMINATION* loci (NIL*DOG*s). The NIL*DOG*s contain introgression fragments of *DOG* alleles of different accessions in the L*er* genetic background, which results in genotypes with different levels of primary dormancy (Figure 1a, bar chart to the right; Alonso‐Blanco *et al.*, 1998; Bentsink *et al.*, 2010). Seeds of the NIL*DOG*s were buried in the soil for 2 years and analysed for both physiological and whole‐genome transcriptomic changes during dormancy cycling. These analyses revealed large transcriptional changes that correlate with previously identified changes in laboratory experiments. In particular, genes related to translation are upregulated in non‐dormant seeds. Our results indicate that transcriptional changes in the endosperm are important for dormancy cycling.

## Results

### Physiology of dormancy cycling

#### Response to the environment during dormancy cycling

To study dormancy cycling at the physiological level, after‐ripened seeds of six genotypes were buried in the soil. After different periods of burial in the field the germination capacity of the seeds was tested in the laboratory. Secondary dormancy was induced in all genotypes (Figure 1a, November 2014–February 2015); however, this process was more rapid in genotypes that possess a high primary dormancy level (NIL*DOG1* and NIL*DOG3*) than in genotypes with a lower primary dormancy level (L*er,* NIL*DOG2* and NIL*DOG22*; Figure 1a). Only NIL*DOG6* seeds, which possess high primary dormancy, showed a secondary dormancy induction similar to the low‐dormancy genotypes during the first autumn and winter. Secondary dormancy is released slower in the more dormant genotypes (April–September 2015). The dormancy cycling pattern is repeated in the following years (2015–2017). NIL*DOG3* shows an aberrant pattern, with lower germination levels in the second cycle (February 2016–February 2017), compared with the first (February 2015–February 2016), and an unexpected low germination in September 2015. As for all genotypes and time points, the viability of the NIL*DOG3* seeds was assessed as described in the Experimental procedures, and the seeds were not found to be dead.

The dormancy cycling pattern followed that of the temperature measured at 5 cm depth in the soil (Figure 1b). We did not observe demonstrable cycling of the moisture content, neither in the soil nor in the seeds, based on measurements using soil moisture sensors, local precipitation and seed moisture content (g H_2_O per g dry weight, gH_2_O gdw^−1^), measured at eight exhumation moments in the second year of the experiment (Figure S1). At 0% relative humidity (RH), Arabidopsis seeds have a moisture content of approximately 0.04 gH_2_O gdw^–1^, at 95% RH, this increases up to 0.4 gH_2_O gdw^–1^ (Basbouss‐Serhal *et al.*, 2015). The moisture contents measured in the field ranged between 0.44 and 1.27 gH_2_O gdw^–1^, which corresponds with fully imbibed seeds (values above 1 indicate that the seeds retained water in the mucilage).

Seed germination after exhumation at one fixed temperature (22°C) allows a direct comparison of the germination capacity of seeds at different time points during the dormancy cycle; however, the response of seeds to the prevailing temperature is likely to provide more insight into responses to the fluctuating environment. This response is important as dormancy cycling is a mechanism to regulate germination in response to the season. Therefore, in the second dormancy cycle, seeds were germinated at both 22°C and at the prevailing outside temperature (Figure 1c). In all genotypes, germinating at the prevailing outside temperature leads to a germination window that is narrower than that at 22°C. This germination window is similar for all genotypes and the highest germination potential is reached between July and September. In L*er*, NIL*DOG1* and NIL*DOG6* genotypes, the optimum germination moment differs between the outside temperature and 22°C. For NIL*DOG3*, the germination is even lower at 22°C compared with the outside temperature.

All genotypes have a period in which there is no germination; however, the depth of the secondary dormancy during this period is likely to be different between the different genotypes. In order to quantify the depth of secondary dormancy, the response to nitrate was tested. Nitrate promotes germination by decreasing ABA levels via the *CYP707A2* hydroxylase (reviewed in Duermeyer *et al.*, 2018). The response to externally applied nitrate was less in the higher dormant genotypes, indicating that secondary dormancy is deeper in these genotypes (NIL*DOG1*, NIL*DOG3* and NIL*DOG6*; Figure 1c).

#### Soil conditions overrule the influence of the maternal environment on dormancy

**Figure 2:**
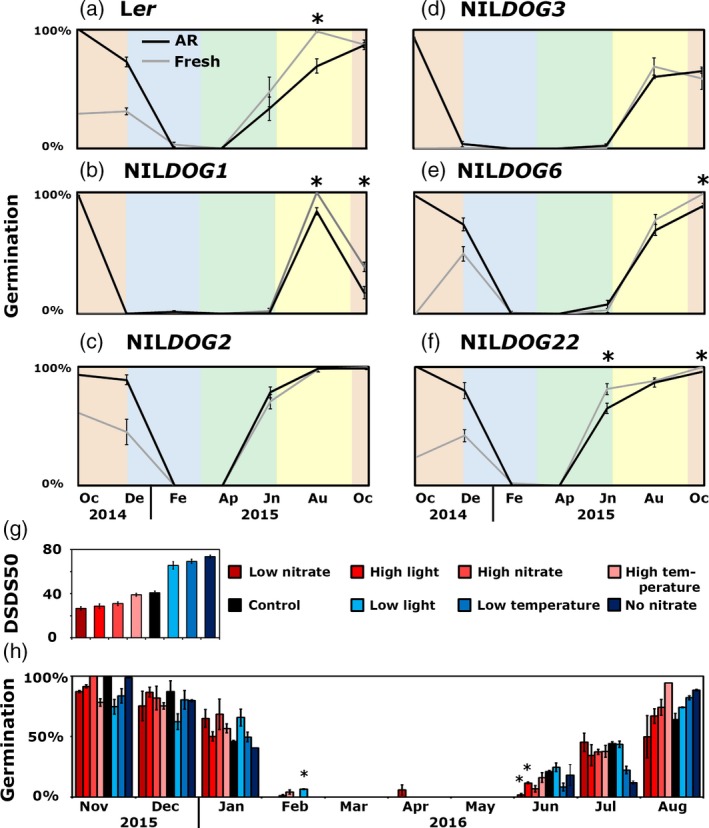
Effect of primary seed dormancy levels on dormancy cycling. (a–f) Germination percentage of fresh (grey) and after‐ripened (AR) seeds (black) after being exposed to field conditions from October 2014 until October 2015. Asterisks indicate significant differences between the fresh and AR seeds (*P* = 0.05, *n* = 4). (g) Days of seed dry storage to reach 50% of germination (DSDS50) of the seeds when grown under different maternal environments. (h) The germination percentages of seeds grown under different maternal environments (colour coded as in g) during burial. Asterisks indicate significant differences by Student’s *t*‐test (*P* = 0.05, *n* = 2, except the August high‐temperature sample, *n* = 1).

The level and rate of secondary dormancy induction correlates with the genetically determined primary dormancy levels. To study the effect of the presence of primary dormancy on secondary dormancy dynamics, freshly harvested (primary dormant) seeds of the six genotypes were buried. Comparing the dormancy induction of the buried fresh and AR seeds, there was no difference observed in the L*er*, NIL*DOG1*, NIL*DOG2* and NIL*DOG3* genotypes (Figure 2a,b,d,e). NIL*DOG6* and NIL*DOG22* showed dormancy release during the first 2 months of burial (Figure 2c,f). The release of dormancy during spring and summer was largely similar between the freshly harvested and AR buried seeds (asterisks in Figure 2a–f indicate significantly different germination percentages). Thus, primary dormancy status does not influence secondary dormancy release in the field.

Next, it was investigated whether soil conditions could also overrule differences in primary dormancy that resulted from different maternal environments during seed production. Hereto NIL*DOG6* seeds developed under different maternal environments (high and low nitrate, low and high temperature, low and high light, compared with control conditions) were buried and seed germination capacity was investigated (Figure 2g). There was no effect of the maternal production environment on the dormancy cycling patterns (Figure 2h). Thus, the primary dormancy status does not continue to secondary dormancy: it was reset by the soil conditions.

#### Secondary dormancy is released faster in the field than by AR

**Figure 3:**
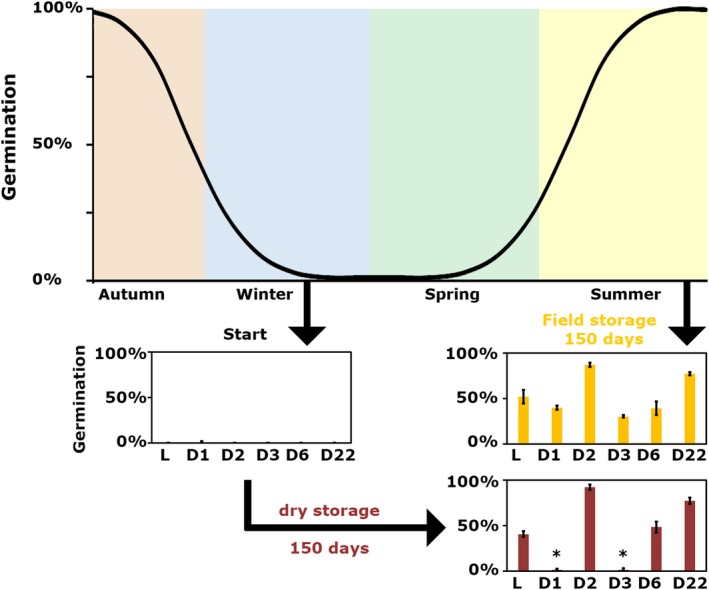
Secondary dormancy release is more efficient in the soil compared with after‐ripening (AR). Fully dormant seeds exhumed in February 2015 were exposed to AR conditions for 150 days. The germination percentage after this AR period was compared with 150 days of field storage. Asterisks indicate significant differences by Student’s *t*‐test (*P* = 0.05, *n* = 3).

Primary dormancy can be released by dry AR. In the soil, where the seeds are moist, secondary dormancy is released when the soil temperatures start to increase. Secondary dormancy in seeds of the very dormant genotype Cvi is released very slowly by AR (Footitt *et al.*, 2011). We wanted to test to what extent secondary dormancy can also be overcome by dry AR, in both low‐ and high‐dormancy genotypes. Therefore, we have stored secondary dormant seeds of the six genotypes that were exhumed in February 2015 for 5 months at 55% RH and 20°C (AR conditions). After 5 months of storage, the low‐dormancy genotypes (L*er*, NIL*DOG2* and NIL*DOG22*) and NIL*DOG6* start to respond to AR treatment, whereas the highly dormant NIL*DOG1* and NIL*DOG3* seeds did not yet respond (Figure 3). These seeds were considered to be viable, based on the absence of fungal growth that usually occurs on dead seeds and because seeds from the same batch were still viable after further field storage (Figure 1a).

### Genome‐wide transcriptional changes during dormancy cycling

#### Large transcriptional changes at dormancy transition phases

**Figure 4:**
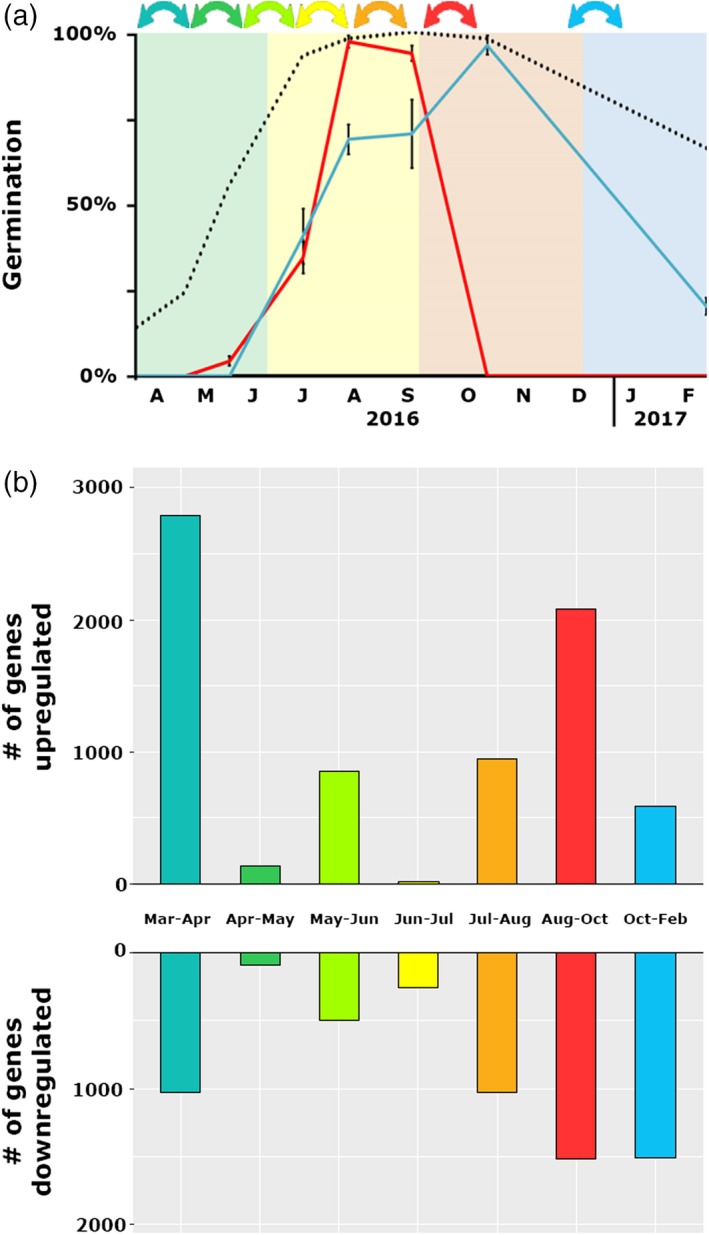
Differentially expressed genes during secondary dormancy cycling. (a) Germination characteristics of L*er* seeds that were buried from March 2016 to February 2017: germination percentages at 22°C (blue), at field temperature (red) and after nitrate treatment (black) are presented. (b) Number of differentially expressed genes between the sequential sampling time points.

To study the genome‐wide transcriptional changes during dormancy cycling an RNA‐seq analysis was performed. RNA was isolated from L*er* seeds at eight storage time points (April 2016–February 2017), almost capturing a full dormancy cycle (Figure 4a). A principal component (PC) analysis on the samples shows that for most time points the biological replicates group together in the first two PCs (Figure S3), with the exception of the samples taken in June 2015. Of these samples one replicate had a lower germination percentage than the other two (18% compared with 49% and 52%). Gene ontology (GO) enrichment analysis of the genes differentially expressed between the individual samples taken in June indicated that the outlier (18% germination) was responding to a biotic stimulus, which suggests that one sample suffered from pathogen attack. Therefore, this sample was excluded from further analyses. PC analysis of all other samples revealed that PC1 explains 46% of the variance, and that PC2 explains 17% (Figure S3b). In general, PC1 separated the samples based on dormancy level.

In total, 10 108 unique genes were differentially expressed between at least two time points (*q* = 0.05, log2 fold‐change, Figure 4b). The largest number of differentially expressed genes (DEGs) between two subsequent time points were found between March and April (2415 upregulated and 856 downregulated genes) and August and October (1677 upregulated and 1241 downregulated genes). These time points reflect switches between dormancy phases: between March and April dormancy release was induced and between August and October dormancy was re‐induced. Pairwise DEGs between all other time points can be found in Table S1.

#### Translation‐related genes are upregulated in non‐dormant seeds

To identify genes that have an expression pattern that follows that of the dormancy cycling, or the inverse, a dominant pattern (DP) analysis was performed (Orlando *et al.*, 2009). The raw counts per gene were used, and after optimization of the analysis 16 DPs were identified (Figure S4). The correlation between the DPs and germination percentages was studied (Figure S4b). Three DPs correlate with the germination capacity of the seeds (DP3, 4 and 14), i.e. the genes are upregulated when the seeds are non‐ or low‐dormant (June–August), this class is referred to as the up–down pattern. The DP containing the highest number of genes was DP3 (1058 genes), for which GO terms related to translation and protein metabolism were enriched (see Appendix S1). DP4 includes 814 genes; however, 466 genes were shared between DP3 and DP4 (as genes were allocated to a DP with a Pearson correlation of <0.9, genes could be allocated to multiple DPs). DP4 has largely the same GO enrichment as DP3. DP14 contained 154 genes (19 genes shared with DP3 and DP4), 72% of which are chloroplastic genes, encoding components of chloroplast ribosomes and polymerases. There was no significant GO enrichment for DP14. When the genes of all three patterns were combined in a GO enrichment analysis, the GO terms for translation and protein metabolism were identified (Appendix S1). No transcription factor motifs were enriched (above a *q* value of 10^−9^) in these up–down patterns, using the tool TF2Network (Kulkarni *et al.*, 2017). There are three DPs (1, 11 and 13) that have a negative correlation with germination, i.e. gene expression decreases when dormancy is released and increases when dormancy is induced, termed the down–up pattern. These DPs do not have enriched GO terms (above a *P* value of 10^−9^), and also not when all genes of these patterns were combined (total 956 genes). A total of 25 transcription factor motifs were found to be enriched, which were recognized by 16 transcription factors (Appendix S1). Fourteen out of these 16 transcription factors were expressed in our data set, and six of these 14 transcription factors have been associated with ABA responses.

#### Effect of large temperature fluctuations on gene expression in the field

The temperature was monitored every 10 min during the whole experiment, which allowed for in‐depth analyses of the effect of temperature on gene expression. Between April and May 2016, there was a dip in temperature followed by a period with higher temperatures. Similarly, there was cold period between July and August 2015, which was followed by a warmer period. These large disturbances in temperature affected gene expression and are reflected in some DPs by dips and peaks. An example of the influence of temperature is DP6, which also follows the germination curve but shows two dips in expression (in May and August 2016). The genes in DP6 show a GO enrichment for ribosomal RNA processing. It seems that the brief periods of increased temperature halt the upregulation of translation‐related genes. Another example is DP10, of which the genes show a peak in expression after both periods of cold followed by warm temperature extremes. The genes in DP10 have GO enrichment for responses to heat.

#### Seeds releasing dormancy in the field show transcriptional patterns that are similar to transcriptional changes in early imbibition

**Figure 5:**
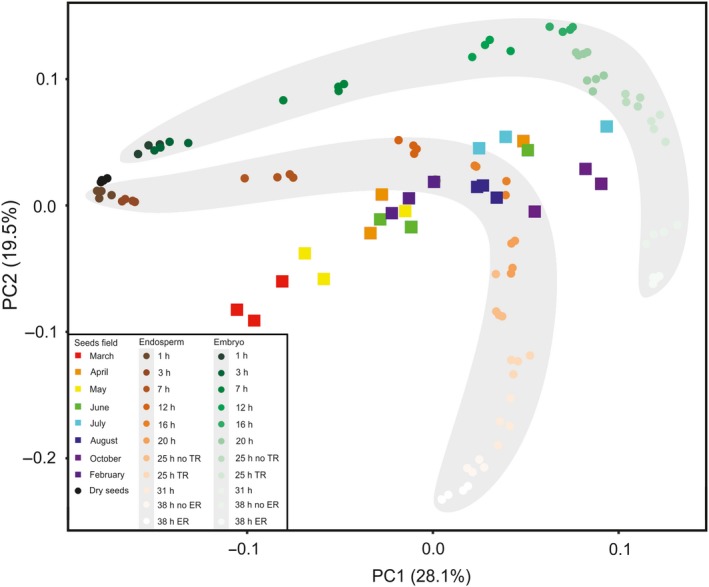
Principal component (PC) analysis of transcriptional changes in seeds that cycle through dormancy compared with those in non‐dormant imbibed seeds. The first two principal components, explaining 28.1 and 19.5% of the variation, respectively, are presented. Square symbols indicate individual samples from the field experiments. Circles indicate individual samples from the Dekkers *et al. *(2013) data set. The grey shaded area containing the brown circles highlights all radicle samples; the grey shaded area containing the green circles highlights all micropylar endosperm samples. The four different quadrants represent clustered samples during early imbibition (top left), late imbibition (top right), germination (bottom right) and secondary dormancy (bottom left).

The transcriptional changes during dormancy cycling were compared with previously identified transcriptional changes during seed imbibition and germination (Dekkers *et al.*, 2013). In this earlier study seeds were separated into four parts, representing the embryonic root (radicle) and hypocotyl, the cotyledons, the micropylar and chalazal endosperm (MCE), and the peripheral endosperm. The radicle and MCE data were combined with the dormancy cycling data in one PC analysis (Figure 5). The biplot of the first two PCs can be separated into four different quadrants, representing clustered samples during early imbibition, late imbibition, germination and secondary dormancy. Moreover, the data reveal that the transcriptomes of the non‐dormant seeds from the field only cluster with the non‐dormant laboratory samples until 25 h of imbibition, which is before the seeds progress towards germination and the testa is ruptured. This is in agreement with no germination being observed in the field experiment. The PCA also shows that the field samples cluster with the endosperm samples, suggesting that transcriptional changes in the endosperm are important for dormancy cycling in the field.

#### Endosperm‐specific genes play a role in dormancy cycling

It is known that the endosperm plays an important role in the regulation of seed dormancy, especially in the signalling for GA and ABA (Lee *et al.*, 2010; Chahtane *et al.*, 2016; Penfield, 2017). To investigate a possible role for endosperm‐specific expression during dormancy cycling we have identified genes that are specifically expressed in the endosperm from the previously mentioned study (Dekkers *et al.*, 2013). Of the 415 endosperm‐specific genes, 161 are expressed (above the threshold of 1.5 fragments per kilobase of transcript per million mapped reads, FPKM, at least at one time point) during dormancy cycling in the field. For these endosperm‐specific genes, whether they cluster to a DP, and if so whether they are significantly over‐ or under‐represented in that DP (compared with all genes in a DP out of all genes expressed in the field; *χ* ^2^, *P* = 0.05, Appendix S1), was calculated. Of these 161 endosperm‐specific genes, 46 clustered to a DP (significant overrepresentation). Of the 46 genes, 23 cluster in the down–up DPs 1 and 11 (for DP1, significant overrepresentation). Eleven genes cluster to DP6 and 9 (for both DPs significantly overrepresentation), in which the expression of the genes shows a strong response to large temperature fluctuations. Eight genes are in the up–down DPs 3, 4 and 14 (significantly under‐represented for DP3; details in Appendix S1). Thus, endosperm genes are generally over‐represented in the DPs.

## Discussion

### Relationship between primary and secondary dormancy depth and dynamics

We showed that secondary dormancy is induced in both high‐ and low‐dormancy genotypes, and confirm that this induction is faster and deeper in the high (primary) dormancy genotypes (Coughlan *et al.*, 2017). Secondary dormancy is induced when seeds in the soil are not able to germinate at times when the environment is not favourable for germination, this includes a lack of light. We buried the seeds at a depth of 5 cm, a depth where non‐dormant seeds did not germinate. In laboratory conditions, however, these seeds germinated without the addition of dormancy‐releasing treatments. In agreement with earlier findings, seasonal dormancy cycling seems to be driven mainly by temperature (Footitt *et al.*, 2011). To identify differences in the depth of dormancy we applied exogenous nitrate (KNO_3_) during the germination assay. Between January and May 2015 nitrate did not trigger germination in the three most dormant genotypes (NIL*DOG1*, NIL*DOG3* and NIL*DOG6*), indicating that the responsiveness to the environment was lower and thus the dormancy was deeper. Although germination rates at 22°C differ a lot between the six genotypes used in this study, the germination at the average prevalent outside temperature was rather similar for all genotypes. This shows the plasticity of the trait, but also how robust its functioning is: in field conditions all genotypes would germinate and establish themselves in the same period (between June and September), despite the genetic differences in dormancy levels.

We observed that alteration of the primary dormancy levels by the maternal environment does not influence secondary dormancy cycling dynamics. This differs from previously reported results (Penfield and Springthorpe, 2012; Auge *et al.*, 2015). In both earlier studies secondary dormancy induction by cold stratification was more efficient in seeds matured at lower temperatures than at warmer temperatures. During these stratification experiments dormancy was first released but then re‐induced after prolonged chilling. It cannot be excluded that the difference in secondary dormancy induction between the maturation temperatures is a result of residual primary dormancy rather than that of the maturation temperature itself. Moreover, differences between our field experiment and the earlier reported laboratory‐based secondary dormancy induction might also be caused by environmental signals other than temperature. The effect of residual dormancy on the induction of secondary dormancy can also explain the lower germination levels of the more dormant genotypes (NIL*DOG1* and NIL*DOG3*) in the second dormancy cycle (October 2015–October 2016), compared with the first dormancy cycle (October 2014–October 2015). When the seeds were buried in October 2014, the seeds had no residual dormancy left, whereas in October 2015 the NIL*DOG1* and NIL*DOG3* seeds had an average germination capacity of 17 and 65%, respectively. These seeds were dormant and viable, not dead. We showed that secondary dormancy can be induced in seeds matured at 20°C when the period of unfavourable conditions (i.e. burial) is long enough (Figure 1a), in contrast to earlier statements (Penfield and Springthorpe, 2012).

A clear distinction between primary and secondary dormancy is not always made regarding the effect of the maternal environment on dormancy levels (Footitt *et al.*, 2017). We hypothesize that the difference of the maternal effect on primary and secondary dormancy (i.e. primary dormancy levels can be altered by the maternal environment but this effect does not last into secondary dormancy cycling) can be explained by ecological function. Primary dormancy can transfer vital information about the environment that the offspring will encounter. If the mother plant experiences less favourable conditions (low nitrate, low light, low temperature) the offspring becomes more dormant (He *et al.*, 2014; Springthorpe and Penfield, 2015). This higher dormancy might postpone the germination to the next year, in which the offspring might encounter a better environment or less competition. For example, low soil nitrate levels indicate that there are competing plants growing in the vicinity (Bewley *et al.*, 2013). Once in the soil the seeds have to keep track of their direct environment, not the preceding maternal environment, in order to germinate at the right moment. Therefore, from an ecological perspective it is reasonable that dormancy levels induced or affected by the maternal environment are reset during dormancy cycling. This might also explain why secondary dormancy is less responsive to AR and cold stratification than primary dormancy (Figure 3), as secondary dormancy release is likely to require different cues.

### Large transcriptional changes at dormancy phase transitions

**Figure 6:**
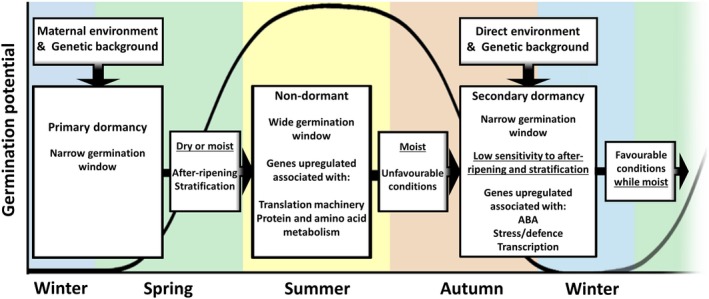
Schematic representation of transcriptomic changes during dormancy cycling. The black line indicates the germination potential during dormancy cycling. In nature, seeds can go through multiple dormancy cycles until they either germinate or lose viability. The gene ontology (GO) enrichment of the upregulated genes for the non‐dormant and dormant stages are derived from the up–down and down–up dominant patterns, respectively. Differentially upregulated genes used for the GO analyses are derived from the pairwise comparison. Full data are given in Appendix S1.

Transcriptome analyses during dormancy cycling in the soil seed bank revealed large transcriptional changes, which on a global scale are similar to transcriptional changes in laboratory experiments (Figure 6). The largest changes occur during the start of dormancy release (between March and April) and the induction of dormancy (between August and October; Figure 4b). During dormancy release in the field, genes associated with the translational machinery are upregulated as well as metabolism and energy‐related genes. This is in accordance with previous observations in the Cvi ecotype, but not in the Burren ecotype (Footitt *et al.*, 2019). This implies that the seeds prepare for germination. This is in accordance with the observation that in seeds stratified in the dark for 48 h (so excluding germination itself), genes associated with RNA processing and the translational machinery are upregulated (Narsai *et al.*, 2011). In seeds from the field there was no initiation of germination, as confirmed by the absence of germination‐specific genes (expressed during the transcriptional phase before testa rupture), previously identified by Dekkers *et al.* (2016). Based on these findings, we conclude that secondary dormancy is overcome in summer and that the seeds prepare for translation, which is required for germination to occur. The regulation of germination is under translational control (Bai *et al.*, 2016), and therefore mRNAs required for the initiation of germination might be transcribed and are ready to be translated when the final trigger for germination, i.e. light, is received. Whether or not there is translation in seeds in the soil seed bank cannot be revealed from this study; however, proteome studies of imbibed dormant seeds show that translation does occur in dormant seeds (Chibani *et al.*, 2006; Bai *et al.*, 2018).

It is known that seeds can survive in the soil for multiple years depending on the species (Long *et al.*, 2014), which might be surprising taking into account the large transcriptional changes that we have identified. Although translation costs much more energy than transcription, the energetic costs of transcription are not to be neglected (Lynch and Marinov, 2015). Seeds are heterotrophic and their survival depends on the reserves that have accumulated during seed maturation. These reserves are required to support the growth of the embryo until the seedling starts photosynthesis and becomes autotrophic. Moreover, part of the energy that is provided by these reserves is consumed by processes that support survival, such as the repair of damaged proteins, RNA and DNA (Waterworth *et al.*, 2016). How dormant seeds manage their resources is currently unknown, but proteome analyses of prolonged imbibed dormant seeds show the downregulation of proteins related to energy and metabolic pathways, which might be a way to avoid wasting energy (Arc *et al.*, 2012).

### The endosperm is important in the perception of the environment

The sensitivity of seeds to temperature fluctuations is shown by the changes in expression following large temperature fluctuations. The DPs that have a peak after strong temperature fluctuations (i.e. in May and August) have enriched GO terms for ‘response to heat’. The possible transcription factors associated with this DP are also identified for the down–up pattern. The patterns that dip in May and August cover genes associated with the translation machinery. In combination, this indicates that such fluctuations in temperature temporarily halt the release of dormancy by re‐inducing dormancy.

Our data suggest that the endosperm can function in the perception of the environment. The fact that a relatively high number of endosperm‐specific genes are in DPs related to response to temperature (DPs 6, 9 and 10) indicates a role for this single‐cell layer in the perception of the environment. A strong role for the endosperm in sensing the environment seems in agreement with the fact that Arabidopsis displays coat‐enhanced dormancy. Coat‐enhanced dormancy is likely to be caused by the endosperm producing ABA signals (Chahtane *et al.*, 2016; Penfield, 2017). The endosperm has been proposed to be important in the perception of the environment in relation to primary dormancy (Dekkers *et al.*, 2016). Genes related to abiotic and biotic stress and hormone signalling were upregulated in the endosperm of imbibed dormant seeds. We hypothesize that temperature fluctuations perceived by the endosperm induce an inhibitory signal to downregulate the genes associated with the translation machinery. It is likely that this inhibitory signal is ABA related, as ABA regulates the upregulation of a set of genes when the seeds are dormant.

### The expression of genes related to secondary dormancy induction differs between field and laboratory induction

The deep secondary dormancy observed in seeds exhumed in winter and the large numbers of DEGs observed during dormancy cycling in the field invites further investigation. A field experiment might not be ideal for studying and validating dormancy cycling mechanisms, however, for practical reasons (laborious and time consuming) as well as legislative reasons (there are strict regulations for field experiments using genetically modified organisms in some countries). We therefore investigated how similar secondary dormancy cycling in the field is to secondary dormancy induced under laboratory conditions. There are a few factors to take into account when comparing these studies. Experimental differences include: (i) secondary dormancy induction conditions , with prolonged dark and cold conditions in the field versus dark and warm conditions in the laboratory (Cadman *et al.*, 2006; Ibarra *et al.*, 2016; Footitt *et al.*, 2017); (ii) dormancy induction is slower and the time between sampling is longer in the field (months), compared with days or weeks in the laboratory; (iii) gradual changes in dormancy levels (in the field) compared with two states, non‐dormant and secondary dormant (in the laboratory) (Ibarra *et al.*, 2016). Genes in which expression is reduced in secondary dormancy in laboratory experiments, such as the GA signalling related genes *REPRESSOR OF GA* (*RGA*), *RGL1*, *RGL2*, *GA INSENSITIVE* (*GAI*) are also significantly reduced in expression during dormancy induction in the field (Ibarra *et al.*, 2016). In contrast, ABA‐related gene expression, i.e. *FUSCA 3* (*FUS3*) and *ABA INSENSITIVE 3* and *5* (*ABI3* and *ABI5*), differed between the field and the laboratory experiments. *FUS3* is not expressed in the field data set, and *ABI3* and *ABI5* expression is stable during dormancy cycling. These three genes show reduced expression during secondary dormancy induction in the laboratory (Ibarra *et al.*, 2016).

The differences could be explained by the comparison of two different dormancy states: the very deep dormancy observed in the field experiment and the shallow dormancy observed in the laboratory experiment. It could also be that in the field, secondary dormancy is a different mix of skotodormancy (dark‐induced dormancy) and thermodormancy (dormancy induced by unfavourable temperatures) than secondary dormancy under laboratory conditions. The conditions used to induce secondary dormancy in the laboratory, osmotic treatments and rapid temperature shifts, are not similar to the dormancy‐inducing conditions in the field. In the field the seeds are fully imbibed and dormancy is induced under prolonged low temperatures, rather than warm temperatures; however, both cold and warm are non‐favourable conditions.

In conclusion, this study shows the relevance of studying (seed) traits under natural conditions, as it uncovered additional aspects of the regulation of secondary dormancy that differ from those found under laboratory conditions. The (confirmation of) a laboratory method that truly mimics secondary dormancy cycling would aid in the further study of secondary dormancy. This study provides insight into the responses of seeds in the context of a natural soil seed bank, in interaction with the environment.

## Experimental Procedures

### Plant material

Six genotypes of *A. thaliana* were used in the experiments: the near‐isogenic lines of the *DELAY OF GERMINATION* loci (NIL*DOG1*, *2*, *3*, *6* and *22*) and the wild type L*er* (Alonso‐Blanco *et al.*, 1998; Bentsink *et al.*, 2010). The plants were grown in a glasshouse according to the methods described by He *et al. *(2014) and harvested in four biological replicates (blocks). After harvest, seeds of each block were divided into two portions with the first stored at −80°C and the second stored under ambient conditions (40–50% RH, 18–22°C). After storing the seeds for 1.5 years at 40–60% RH and 20–22°C or −80°C, respectively, seeds from both conditions were buried. Before burial, the seeds from the freezer were stored on the bench for 3 days to recover. These seeds were termed ‘fresh seeds’, as they did not experience any dry AR. The seeds that were stored on the bench were termed ‘AR seeds’. For both fresh and AR seeds, a single block did not contain enough seeds. Therefore, the seeds of three blocks of one genotype were mixed together (with fresh seeds and AR seeds kept separate). The mixed seeds (blocks 1, 2 and 3) were divided into four batches, which were separately buried as biological replicates. The AR seeds were exhumed every month over a period of 2 years (from November 2014 to October 2016, and in February 2017), fresh seeds were exhumed every 2 months over a period of 1 year (from December 2014 until October 2015).

The NIL*DOG6* seeds used here were developed in different maternal growth environments (high and low nitrate, low and high temperature, low and high light, compared with control conditions), as described previously (He *et al.* 2014). After seed production in the different maternal environments, seeds were stored at ambient conditions (20–22°C, 40–60% RH) for 1.5 years. Two biological replicates were used for the burial experiment.

### Sample preparation for burial

Approximately 500 seeds per replicate were mixed with 12 g of glass beads (50–75 μm in diameter; ThermoFischer Scientific, https://www.thermofisher.com), according to the method described by Footitt *et al.* (2011). This mixture was placed in a nylon mesh bag (6 cm × 6 cm, gauze of 125 m, sewn with nylon thread) containing a small plastic label. The bag was closed with a plastic paper clip (Advantus Plastic Clips; https://shopadvantus.com). All materials mentioned above were chosen to endure long‐term burial and to release no nutrients or chemicals of any kind.

### Burial and monitoring soil conditions

The seeds were buried in mid‐October 2014 in an open field (Unifarm, Kielekampsteeg, Wageningen, the Netherlands) as described by Footitt *et al.* (2011), with some alterations. The seeds were not treated with fungicide or anything of the sort before burial. Thirty 5‐cm‐deep patches were dug into the ground and separated by wooden planks. Each patch measured 50 cm × 75 cm. In each patch one exhumation was placed, consisting of 24 bags (with six genotypes and four technical replicates). The bags were then covered by 5 cm of heat‐sterilized soil (sandy clay). During the experiment, the temperature and soil water activity were measured at 5 cm depth with the following equipment: logger, model DT85 (DataTaker; ThermoFisher Scientific, https://www.thermofisher.com); temperature, T thermocouple (copper/constantan) (Tempcontrol, https://www.tempcontrol.nl); soil water activity, Watermark soil moisture sensor (Eijkelkamp). To support these soil moisture measurements, which suffered from defective sensors during certain periods (Figure S1), precipitation data and the temperature under 5 cm of bare soil obtained from the nearby De Veenkampen weather station (Veensteeg, chair group of meteorology and air quality, Wageningen University, 2.5 km away) were taken along with the analyses. Soil water suction was recorded in the first year by measuring the water activity of the soil. The second year, the moisture content of the buried seeds (buried solely for this purpose) was measured, by dry weight (Figure S1).

### Seed exhumation and retrieval

The seeds were exhumed with the aid of a custom‐made box, with two holes with gloves attached (Figure S1). The box allowed the seeds to be taken from the soil and placed in a light‐proof container while in the dark. The seeds were transferred in the light‐proof container to a dark room with green light. The seeds were washed from the bags with demineralized water at room temperature according to the protocol described by Footitt *et al.* (2011). In detail, the content of the bags was rinsed into a 50‐ml falcon tube with demineralized water. Once the seeds and beads had settled at the bottom, the water was then poured off. This removed most of the sand and debris. Then demineralized water was again added. Care was taken not to rinse or shake the mixture too much, as this might damage the seeds. Seeds, being lighter than the beads, float on top of the beads. The seeds were then collected by pipetting with a cut‐off 200‐µl tip into an Eppendorf tube. Superfluous water was removed with a pipette. After washing, the germination capacity of approximately 50 seeds was investigated by a germination test. The other seeds were dried under 40% RH in the dark at room temperature (20–22°C). After approximately 30 h of drying, the seeds were transferred either to ambient conditions (November 2014–February 2016) or to −80°C storage (March 2016–February 2017). Germination tests were performed after drying, and the drying did not alter the germination and physiology of the seeds (Figure S2).

### Germination and viability assays

For all germination experiments, seeds were sown on blue germination paper in trays with 48 ml of demineralized water and placed in a cabinet at 22°C with continuous light. Each tray contained six samples of approximately 50 seeds. Seed germination was followed for 5 days using the Germinator system (Joosen *et al.*, 2010). For all germination experiments, the viability of the non‐germinated seeds was checked by placing the seeds in a new germination tray with 10 mm KNO_3_ added to the demineralized water. After 1 day in nitrate the seed coat was removed from the remaining non‐germinated seeds. At 2 days after seed‐coat removal, viability was assessed by checking for growth of the embryo (greening of the cotyledons and radicle elongation).

### RNA sequencing and library construction

RNA sequencing was performed on samples from eight time points (AR samples, three replicates, April 2016–February 2017). RNA was isolated from approximately 150 seeds using the Hot Borate protocol (Wan and Wilkins, 1994). RNA quality and quantity was assessed with the Trinean Xpose (now sold as Lunatic, https://www.unchainedlabs.com). Preparation of the libraries and Illumina sequencing was performed by the in‐house sequencing facility. RNA (500–1000 ng) was used for poly‐A library preparation. The sequencing length was 50 nt and the read library was single end.

### Read alignment and transcript assembly

Read alignment was performed using Araport11 (Cheng *et al.*, 2017) as the reference genome sequence in hisat2 2.0.5 (Kim *et al.*, 2015). Transcript assembly, quantification and splice variants were determined using StringTie (https://ccb.jhu.edu/software/stringtie, Pertea *et al.*, 2015). The annotation and statistical analysis were performed using the bioconductor package ballgown (Frazee *et al.*, 2015), in the programming language r (R Development Core Team, 2009). Expression levels were described using fragments per kilobase per million reads sequenced (FPKM) to normalize for sequencing depth and gene length, and were log transformed.

### Comparative principal components analysis (PCA)

The microarray data for non‐dormant Col‐0 seeds was taken from Dekkers *et al*. (2013). In brief, the seeds were sampled and dissected over the course of imbibition and germination and microarray analysis was performed to quantify gene expression. The normalized microarray data and the RNAseq data from this study were scaled and centred separately using the r scale function, and then all two data sets were combined into one PCA analysis using the r prcomp function.

### Dominant pattern analysis

Dominant pattern (DP) analysis was performed using the script described by Orlando *et al.* (2009). The method includes two rounds of sequential clustering. First, a variant of the K‐means method, fuzzy K‐means, separates the large data set into preliminary groups. From these groups, initial patterns are generated and a probability cut‐off variable is used to assign the genes to a pattern. This is followed by single‐linkage hierarchical clustering of this initial set of patterns. The resulting tree is cut at the pattern similarity cut‐off, set to a default of 0.1, so that all patterns with a Pearson correlation of 0.9 or above are clustered and collapsed together. Finally, genes are assigned to the patterns based on their distance to each pattern (based on the Pearson correlation). K‐choice (the number of initial clusters) was set at 25. The probability cut‐off was generated automatically by the algorithm. Two rounds of pre‐clustering filtering were applied: a median absolute deviation (MAD) threshold of 0.5 was used to remove genes with little variance in their expression and then a filter removed all genes with a maximum FPKM value of <1.5.

### Differentially expressed genes

The edger package was used for the DEG analysis (Robinson *et al.*, 2010). Before analysis, the data were filtered to remove genes for which no samples had values >1.5 FPKM and a number of reads >10. Of the 37 336 genes, 17 153 remained for the analysis, which was done using the raw reads and the glmqlftest function in r.

### Gene ontology enrichment analysis

The GO enrichment analysis was performed with the online tool Gorilla (http://cbl-gorilla.cs.technion.ac.il/; Eden *et al.*, 2009). The unranked approach using a target list (e.g. genes of one DP) and a background list, consisting of all genes that were expressed above 1.5 FPKM at least at one time point, was used.

## Conflict of Interest

The authors declare no conflicts of interest.

## Author Contributions

LB, GB and HN designed the experiments. GB performed the field experiment. GB and HN performed the transcriptome experiment. GB, AV and HN conducted the analyses. GB and LB drafted the article. All authors participated in revising and editing the article.

## Supporting information


**Figure S1.** Precipitation and temperature data from the burial experiment.

**Figure S2.** Germination of the field samples before and after drying.

**Figure S3.** Principal component analysis based on gene expression data.

**Figure S4.** The 16 dominant patterns identified.

**Table S1.** DEGs between every time point during dormancy cycling in the field.

**Appendix S1.** GO enrichment and dominant pattern analysis.

## Data Availability

The sequence data have been submitted to the NCBI Sequence Read Archive under project number PRJNA554414.
