# Supplementary material for: Dormancy cycling: translation‐related transcripts are the main difference between dormant and non‐dormant seeds in the field
Source: Plant J. 2020 Feb 5;102(2):327–39. doi: 10.1111/tpj.14626 (PMC7217185; doi:10.1111/tpj.14626)
Supplement: Supplementary file 1 — Figure S1. Precipitation and temperature data from the burial experiment. [file TPJ-102-327-s001.docx]

**Figure S1. The precipitation and temperature data during the burial experiment.** A) Temperature (measured at 5 cm below the surface) and precipitation data during the field experiment, data from weather station de Veenkampen). B) The measurements from the sensors and (D) interpretation of the values. C) The moisture contents (g H_2_O g dw^-1^) measured during the field experiment. The dashed line represents the maximum moisture content measured at 95% RH (Basbouss-Serhal *et al.,* 2015). E) Visualisation of the soil with corresponding soil water suction. F) The box used for dark exhumation.

**
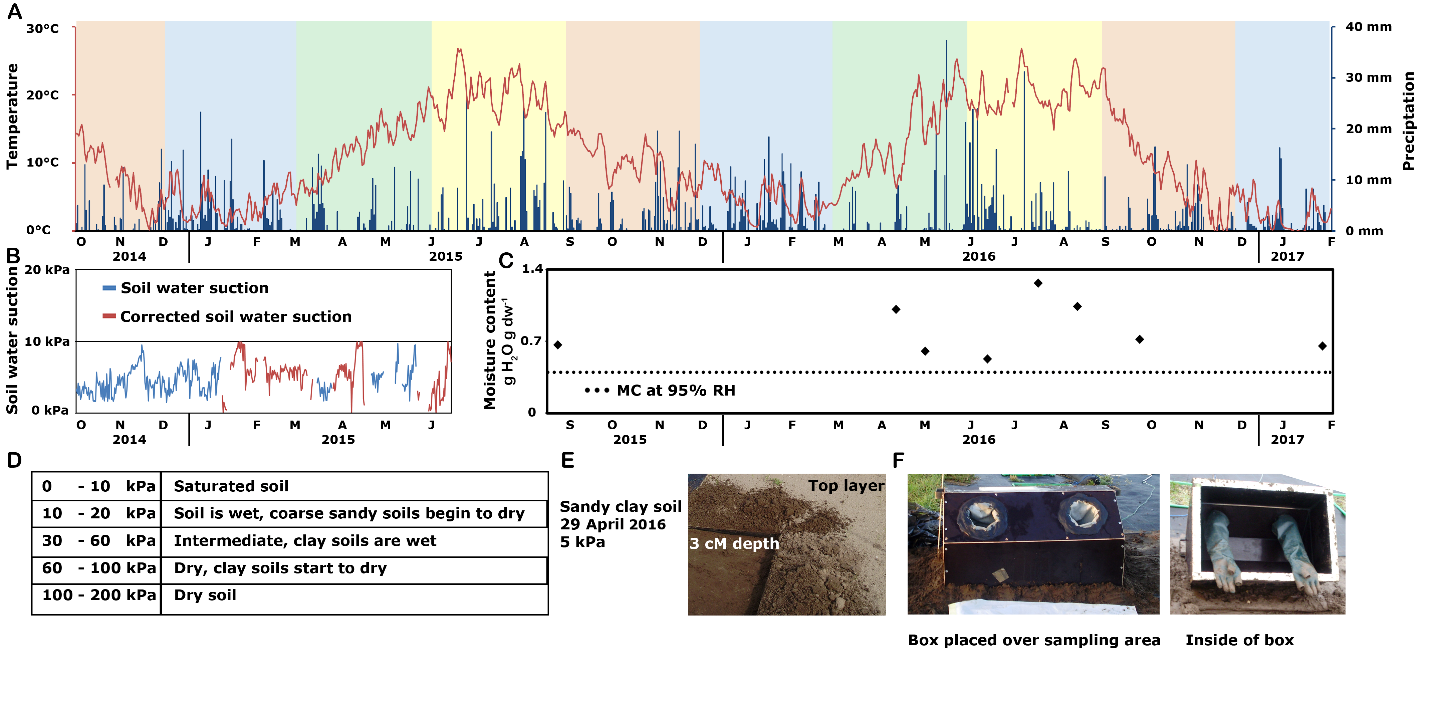
**
