# Supplementary material for: Dormancy cycling: translation‐related transcripts are the main difference between dormant and non‐dormant seeds in the field
Source: Plant J. 2020 Feb 5;102(2):327–39. doi: 10.1111/tpj.14626 (PMC7217185; doi:10.1111/tpj.14626)
Supplement: Supplementary file 2 — Figure S2. Germination of the field samples before and after drying. [file TPJ-102-327-s002.docx]

**Figure S2. Effect of drying on the germination.** Germination of the six genotypes before (blue line) and after drying (orange line) (n=4). The germination before drying is the germination directly from the field at 22°C, and is the same data as shown in Figure 1A. The seeds were dried after removal from the bags at room temperature (20-22°C) in the dark for *c.* 30 hours.

**
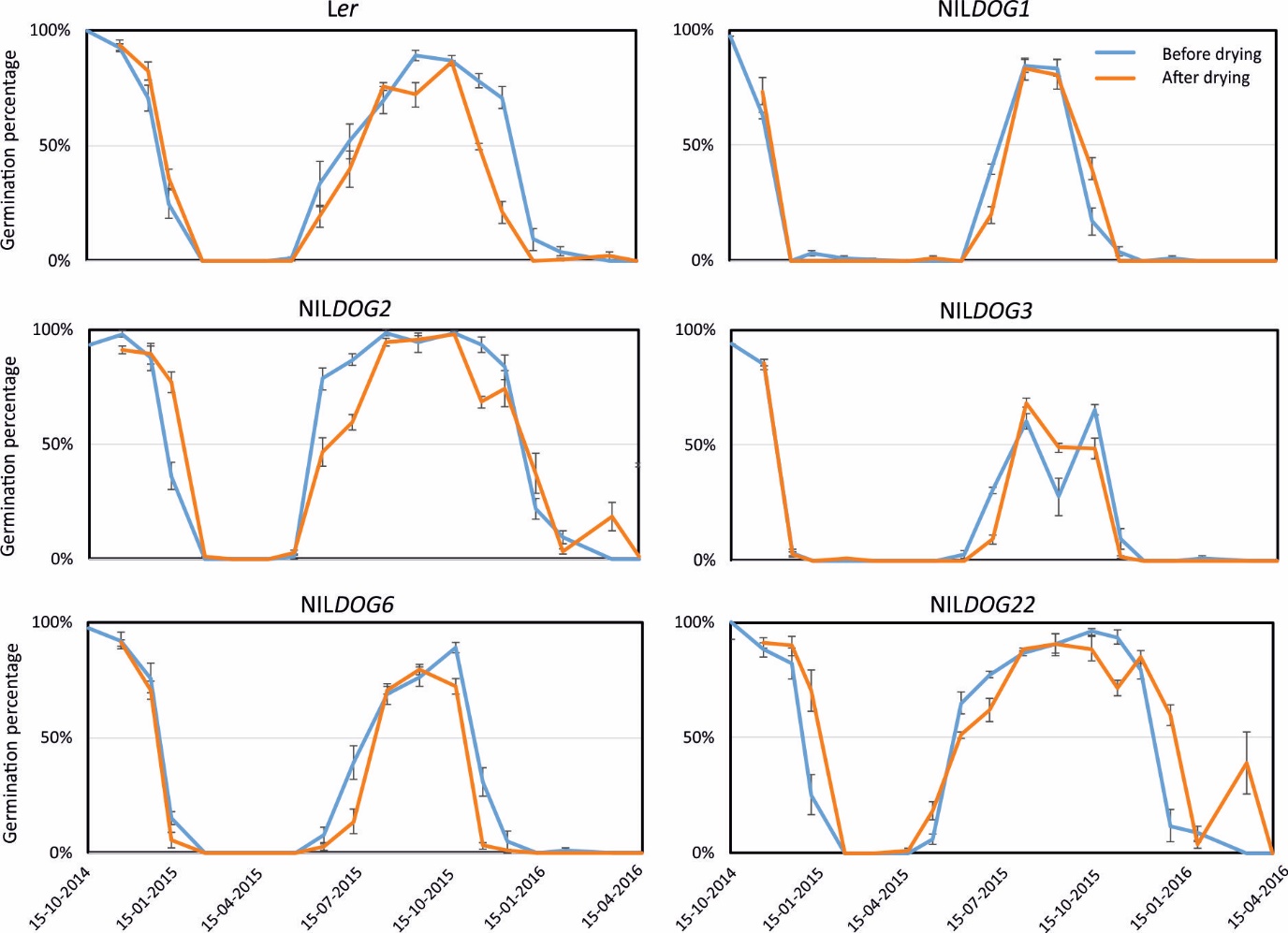
**
