# Supplementary material for: Dormancy cycling: translation‐related transcripts are the main difference between dormant and non‐dormant seeds in the field
Source: Plant J. 2020 Feb 5;102(2):327–39. doi: 10.1111/tpj.14626 (PMC7217185; doi:10.1111/tpj.14626)

**Figure S3. Principal component analysis based on the gene expression data.** A) PCA plot of the individual samples. Numbers indicate individual germination percentages. B) The average germination at 22°C (solid black line) and after nitrate treatment (dashed line). The coloured signs correspond with the legend in A.


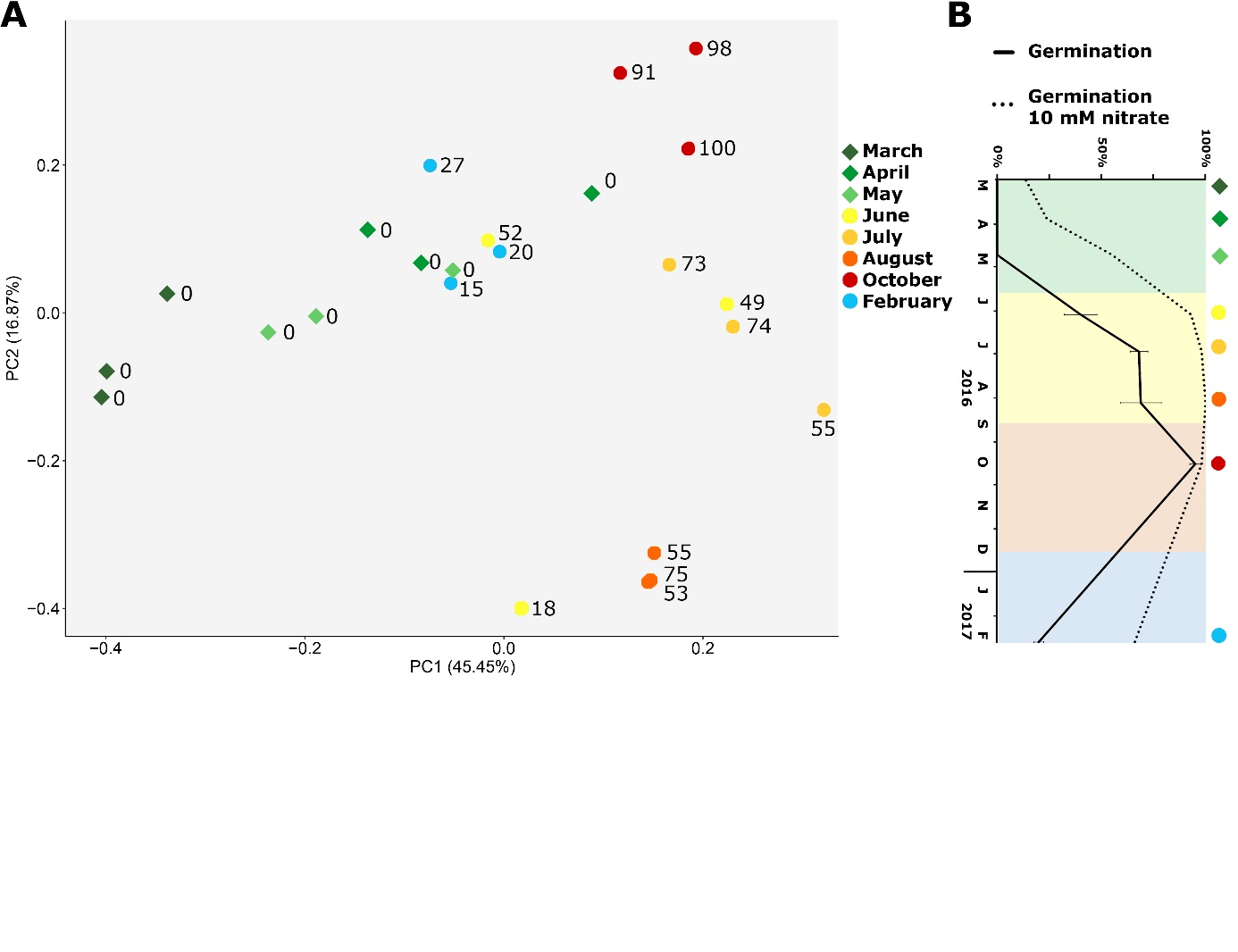

Supplement: Supplementary file 3 — Figure S3. Principal component analysis based on gene expression data. [file TPJ-102-327-s003.docx]
