# Supplementary material for: Dormancy cycling: translation‐related transcripts are the main difference between dormant and non‐dormant seeds in the field
Source: Plant J. 2020 Feb 5;102(2):327–39. doi: 10.1111/tpj.14626 (PMC7217185; doi:10.1111/tpj.14626)
Supplement: Supplementary file 4 — Figure S4. The 16 dominant patterns identified. [file TPJ-102-327-s004.docx]

**Figure S4. The 16 dominant patterns identified.** The blue line shows the DP (on an arbitrary scale). The grey line is the germination at field temperature, the dashed grey line germination at 22°C and the dotted grey line germination after nitrate and at 22°C. The grey shade represents the field temperature. The number of genes that cluster to the pattern are indicated. The table shows the Pearson correlation between the germination pattern and the DPs. Discussed correlation are highlighted in green (positive correlation) and in red (negative correlation).


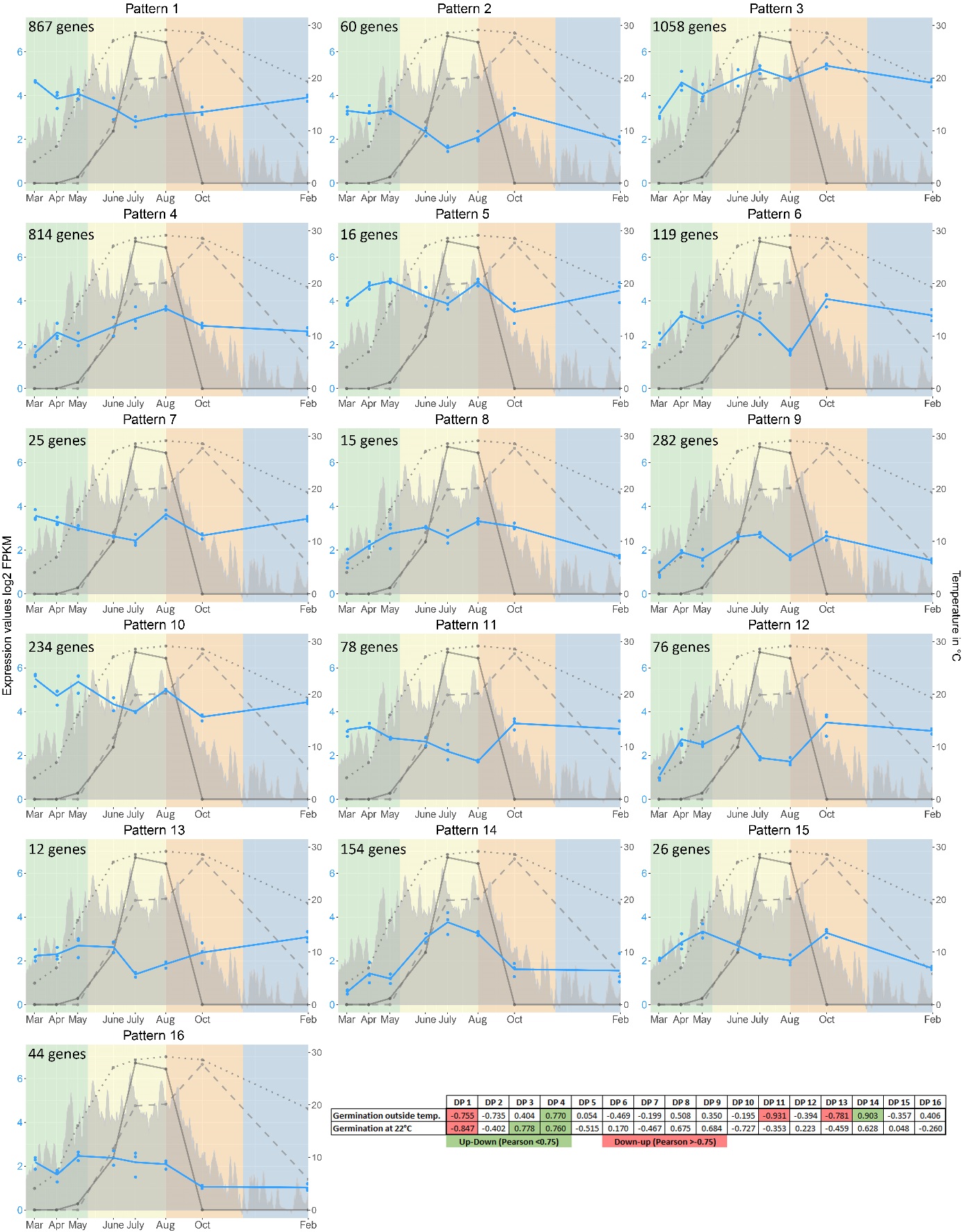


A

B
