# Supplementary material for: Dormancy cycling: translation‐related transcripts are the main difference between dormant and non‐dormant seeds in the field
Source: Plant J. 2020 Feb 5;102(2):327–39. doi: 10.1111/tpj.14626 (PMC7217185; doi:10.1111/tpj.14626)
Supplement: Supplementary file 5 — Table S1. DEGs between every time point during dormancy cycling in the field. [file TPJ-102-327-s005.docx]

**Table S1.** DEG between every time point during dormancy cycling in the field. (q value < 0.05, 2 log fold change).

|  | Up-regulated | | | | | | | | |
| --- | --- | --- | --- | --- | --- | --- | --- | --- | --- |
| Down-regulated |  | March | April | May | June | July | Sept. | Oct. | Feb. |
|  | March | **X** | 2415 | 1550 | 3179 | 3760 | 3372 | 3710 | 2118 |
|  | April | 856 | **X** | 57 | 185 | 785 | 993 | 532 | 18 |
|  | May | 505 | 34 | **X** | 531 | 1788 | 1595 | 1590 | 214 |
|  | June | 1783 | 149 | 391 | **X** | 2 | 400 | 216 | 175 |
|  | July | 2904 | 1289 | 1925 | 63 | **X** | 739 | 768 | 1230 |
|  | Sept. | 2481 | 1399 | 1640 | 850 | 778 | **X** | 1677 | 1152 |
|  | Oct. | 2508 | 557 | 1333 | 254 | 367 | 1241 | **X** | 480 |
|  | Feb. | 1228 | 256 | 674 | 648 | 1395 | 1335 | 1125 | **X** |
